# Food supplied to Gaza during seven months of the Hamas-Israel war

**DOI:** 10.1186/s13584-025-00668-6

**Published:** 2025-02-12

**Authors:** Naomi Fliss-Isakov, Dorit Nitzan, Moran Blaychfeld Magnazi, Joseph Mendlovic, Sharon Alroy Preis, Gilad Twig, Aron M. Troen, Ronit Endevelt

**Affiliations:** 1 Department of Health Promotion, School of Public Health, Medical and Health Sciences, Tel Aviv University, Tel Aviv, Israel; 2 The Gertner Institute for Epidemiology & Health Policy Research, Sheba Medical Center, Ramat Gan, Israel; 3 Israel Ministry of Health, Public Health Services, Jerusalem, Israel; 4 Master’s Program in Emergency Medicine, School of Public Health and Food Systems, One Health and Resilience Research Center (BGU-FOR), Ben Gurion University of the Negev, Beer Sheva, Israel; 5 Department of Pediatrics, Shaare Zedek Medical Center, Hadassah University School of Medicine, Jerusalem, Israel; 6 Division of Endocrinology, Diabetes and Metabolism, Sheba Medical Center, Ramat Gan, Israel; 7 Department of Preventive Medicine and Epidemiology, School of Public Health, Medical and Health Sciences, Tel Aviv University, Tel Aviv, Israel; 8 Institute of Biochemistry Food and Nutrition Science, The Robert H. Smith Faculty of Agriculture Food and Environment, The Hebrew University of Jerusalem, Rehovot, Israel; 9 Faculty of Welfare and Health, School of Public Health, University of Haifa, Haifa, Israel

**Keywords:** Hamas-Israel war, Food insecurity, Famine

## Abstract

**Background:**

The ongoing Hamas-Israel war has put the civilian population in Gaza at risk of severe food and nutrition insecurity. Our goal was to provide objective, verifiable data to ascertain amounts and nutritional content of food supplied to Gaza through Israeli border crossings from January to July 2024. We aimed to assess their compliance with Sphere international humanitarian standards for food security and nutrition maintenance in crisis affected populations.

**Methods:**

We obtained the registry of all food supplied to Gaza via air drops and land crossings from Israel’s Coordinator of Government Activities in the Territories (COGAT) from January to July 2024. This registry itemizes daily food shipments, their items and estimated weights. Food items in shipments were categorized, quantified, assessed and summed for energy (kcal), protein (g), fat (g), iron (mg) content, based on food labels and composition tables. We then calculated supply per capita per day supplied to the the Gaza Strip, according to the most recent population census of Gaza. Finally, we compared it to the Sphere standards for population food security.

**Results:**

Adjusting for projected food losses, a net total of 478,229 metric tons of food was supplied to Gaza over the seven consecutively studied months. The average amount of energy available per person per day was 3,004 kcal, with 98 g of protein (13% of energy), 61 g of fat (18% of energy), and 23 mg of iron. Except for February, when entries dropped from January, there was a steady increase in the tonnage, energy, macronutrients and iron content of donated foods supplied to Gaza registered by COGAT. The amounts of energy, protein, and fat, but not dietary iron, in food crossing the border into Gaza consistently exceeded Sphere standards after making conservative adjustment for high food loss and the age distribution of the Gazan population.

**Conclusions:**

This study assessed food deliveries by type, amount and nutrient composition, supplied to Gaza from January to July 2024. We found that, except in February, food crossing the borders into Gaza exceeded per capita minimal requirements for humanitarian aid. While reliable data do not exist for critical dimensions of food access and consumption across Gaza, these estimates suggest that adequate amounts of nutritious food were being transported into the Gaza Strip during most of the 1st half of 2024. We propose that with increased cooperation of all partners and shared efforts to overcome barriers, communication and data sharing, the UN Food Security Cluster, COGAT and humanitarian assistance agencies can develop a comprehensive, continually updated database to gauge food availability, access, nutritional value, and gaps to address, especially in the areas most disrupted by conflict in Gaza. This will also help ensure that food donations supplied to Gaza reach populations in greatest need.

**Supplementary Information:**

The online version contains supplementary material available at 10.1186/s13584-025-00668-6.

## Introduction

This study assessed the food supplied to the Gaza Strip – a necessary initial condition for meeting the nutritional needs of the Gazan population from January to July 2024. The war between Hamas and Israel began on October 7, 2023 when Hamas launched a massive, coordinated terrorist attack on civilian communities in southern Israel. Over 1,200 civilians, including babies, women, and elderly people, were murdered, and hundreds were abducted into Gaza [1–3],

The Israel Defense Forces (IDF) entered Gaza on October 27, 2023, as part of a military operation, while coordinating the flow of international humanitarian assistance to the Gazan population. Israel maintains a dedicated unit called COGAT (Coordinator of Government Activities in the Territories), with responsibility to coordinate humanitarian efforts in the Gaza Strip by the international agencies and the private sector in Gaza. These efforts include facilitating the entry of donated humanitarian aid and commercial food, and reviewing routes used for distribution of aid and convoys [4]. The population in northern Gaza was advised by the IDF to move to designated humanitarian areas in the southern Gaza strip. Population displacement, extensive infrastructural damage, and constraints on the delivery and distribution of food in the ensuing combat operations, precipitated a critical war crisis in food insecurity. Fighting during November and December caused rapidly deteriorating humanitarian conditions [5]. UN humanitarian agencies, including the Office for the Coordination of Humanitarian Affairs (OCHA), The World Food Program (WFP), The United Nations Children’s Fund (UNICEF), The United Nations Relief and Works Agency for Palestine Refugees in the Near East (UNRWA), The Food and Agriculture Organization (FAO) and The World Health Organization (WHO), were involved in purchasing, transporting, delivering, and coordinating the distribution of humanitarian assistance under the framework of the Food Security, Nutrition and Health Clusters. They issued several reports based on the Integrated Food Security Phase Classification (IPC) system that led to warnings of famine in Gaza if more preventive measures were not immediately taken [6–8], particularly in the northern governorates of the Gaza Strip where famine was projected between mid-March and May 2024 [8]. The report implicated Israel in obstructing the delivery of humanitarian aid, noting a constricted flow of humanitarian and commercial imports far below dietary requirements that would cause the food availability situation to worsen with continued restrictions on food imports [7]. The projection, widely disseminated in the media [9–12], heightened concerns in the scientific literature [13–16] that Israel was using starvation as a weapon of war, based on reports of few food trucks crossing into the Gaza strip shortly after the war began, especially as Gaza was heavily dependent on food aid prior to the war [17].

The Government of Israel’s position, as stated by COGAT, has been that from early in the war it has put no restrictions on admitting humanitarian aid into Gaza as long as it passes security screening [18], prohibiting only trucks carrying items that could be used for terror activities.

Documentation and records of food transport were scant during the early months of conflict. Beyond concerns about enough food being available early in the war, there were also no reports on whether the nutritional value of food supplied to Gaza was sufficient to meet the population’s needs. These circumstances motivated this study which has focused on the quantity and quality of food provision to Gaza based on reliable, accurate and comprehensive shippingrecords which became available starting January 2024.

We relied on the records of Israel’s COGAT, the government agency responsible for coordinating and ensuring the safe passage of humanitarian aid and workers into Gaza [19]. Food commodities evaluated in this report were donated by international donors and aid agencies (the food security clusters) who are responsible for obtaining and distributing the food throughout Gaza during this time of war.

As a first step toward evaluating food availability and security in Gaza, we have sought to investigate the quantity and quality of food donated and transferred by the food security cluster partners, and private sector, into Gaza through Israeli borders [19], based on operational records maintained by COGAT from January through July 2024. We used international humanitarian standards to assess whether the aid met the Gazan population’s requirements for energy, protein and iron, as a sentinel micronutrient. We did not have access to reliable, transparent and independently verifiable data on delivery and storage of food throughout the territory, access to food through markets or distribution centers, within Gaza [20, 21]. Thus, we focused on accessing, analyzing and critiquing data in verified documents on food supplied into the Gaza Strip during the war.

## Methods

### The COGAT food transfer database

Beginning in December 2023, COGAT established a registry documenting the food donation transfer requests, and thier clearance status [22]. The database, which was provided to the authors by COGAT, lists the date of shipment, the donor/source of supply, the weight (metric tons), and the food items transferred through the border crossings into Gaza. Importantly, this database includes data on the vast majority of food that entered the Gaza Strip. We analyzed all food shipments registered and authorized by COGAT, and delivered by land and air, across the border from January through July 2024. Shipments which were not authorized, and which did not actually enter Gaza were not included in this analysis. The COGAT online dashboard (https://gaza-aid-data.gov.il/main/) publishes the core data that we analyzed as a freely downloadable file, listing individual shipments by date, their route of entry (e.g. by land, air, or sea) classification (e.g. food, medical supplies, shelter, mixed goods, etc.), the donor, and shipment weight. We were further supplied with the exact content of each shipment carrying food.

COGAT manually verifies the contents of all trucks at the border crossing points and lists the contents and shipment weights in its database. In some cases, the weight of food content is provided by the donors and recorded by COGAT. In cases when the weight of food on trucks was not provided by the donor, the weight of food shipment was estimated based on the average weight of shipments of the specific content, donor and truck size, when those were provided by donors. According to these data, the following tonnage was applied: trucks carrying food (excluding wheat flour) from UN agencies − 15 tons; trucks carrying food (excluding wheat flour) from all other sources − 20 tons; trucks carrying wheat flour from all sources – 30 tons; and trucks carrying a combination of food and other aid supply – 15 tons.

A few other differences exist between the data in the COGAT dashboard and our data: the COGAT database details the weight of the food in the shipments *before* adjusting for food loss, and it dose not include the weight of trucks with “mixed” cargo in the count of shipments or weight (Tons) of food. Thus, the gross weights of food listed on the COGAT dashboard are different from those that we present in this manuscript.

### Supply classification

We classified the food consignments for each delivery as follows, and list them in Table 1:

1. *Specific food commodities*: deliveries that included specific foods. The nutritional content contribution of each food item in the parcels was calculated based on the United States Department of Agriculture (USDA) database [23] as depicted in supplementary Table 1.
2. *Standardized food parcels*: shipments of food parcels from the UN and other humanitarian aid agencies that provide a detailed description of their food content. These include food parcels donated by the International Federation of the Red Cross and Red Crescent Society (IFRC ICRC), World Food Program (WFP), OXFAM, The United Nations Relief and Works Agency for Palestine Refugees in the Near East (UNRWA), as well as the World Central Kitchen (WCK), and others. The nutritional contribution of each food item in the parcels was calculated based on the USDA database. The nutritional values per 100 g were calculated for gross dry food weight, as described in supplementary Table 2.
3. *Non-standardized food parcels*: shipments including a large variety of food commodities in unstandardized combinations (for example, a truck carrying rice, cheese, canned legumes, watermelon, and onions). Since these deliveries varied in food combinations, we accounted for their nutritional content as the weighted mean of the nutritional values of all food commodities delivered into Gaza during the time of the study, in trucks categorized as specific food commodities (supply category 1). The nutritional contribution of each food item delivered as specific food commodities was calculated based on the USDA database (supplementary Table 1).
4. *Mixed food parcels*: shipments listing standard and/or nonstandard food parcels together with non-food items (such as clothing, medical, or hygiene supplies). We accounted for the nutritional content of these deliveries, similarly to standardized and non-standardized food parcels (supply categories 2 and 3). Their contribution to nutrient availability to shipments stem from the adjustment of weight of food in the shipments.
5. *Cooked meals*: shipments containing cooked meals. Since most cooked meals were donated during the month of Ramadan, we estimated the contents of cooked meals in deliveries based on prior knowledge of the traditional Ramadan evening meal. Our calculated meal is based on a combination of cooked grain, cooked meat in sauce, bread, two forms of spread, and dessert. The nutritional contribution of each food item in the parcels was calculated based on the USDA database (supplementary table 3).
6. *Food items for infants*: shipments which include baby food, nutritional supplementation and infant formula from 6 to 12 months of age. The nutritional contribution of each food item in the parcels was calculated based on the USDA database and commercial product food labels. Importantly, we based our assumption of the kind of infant formula in food deliveries on United Nations Children’s Fund (UNICEF) and World Health Organization (WHO) guidelines, by which food aid which is destined for distribution among the general population should not include formulas for infants under six months of age. This prohibition is based on health and food safety concerns, including lack of access to water, hygiene and sanitation in times of crisis. Since most of the food in this category was donated by UN organizations, we cautiously assumed it adhered to their specifications, recognizing exceptions were possible during conflict. Therefore, food deliveries recorded to contain “infant formula” were considered for age > 6 months, and their nutritional content was assessed accordingly.

### Food weight adjustment

While there is no standardized method to adjust for potential food loss in humanitarian shipments, we adopted the common practice of using a 15% food weight loss adjustment factor for food commodities, non-standardized food parcels, cooked meals and infant foods. We arrived at this value in consultation with international humanitarian aid experts involved in the issue who wished to remain anonymous due to the sensitivity of the matter (personal communication). In the case of standardized food parcels, we calculated the weight adjustment factor based on the standard IFRC food parcel. This parcel weighs 12 kg in total, while the dry food weight amounts to 7 kg. Therefore, we adjusted the weight of standardized parcels based on 40% loss. In cases of mixed shipments including food parcels, and other aid (such as clothing medical and hygiene supplies), we adopted a conservative approach, adjusting the net weight of food to 50% of the consignment’s weight, and made further adjustments for food of 40% or 15% respectively, if parcels were standardized or non-standardized (Table 1).

### Estimated nutrient composition of shipment content

We estimated the energy (kcal/ton), protein (g/ton), fat (g/ton), and iron (mg/ton) content of each donated, approved shipment that passed into Gaza, according to the food composition values. (Table 1, see Supplementary Tables 1, 2 and 3 for details). We chose to assess energy and protein availability due to their essentiality in maintaining normal weight and body composition and treating malnutrition [24].We assessed fat as an energy source and in facilitating absorption of fat-soluble vitamins, and iron as a crude proxy for nutrient-dense animal source foods (such as meat, chicken, fish, and eggs), since iron-deficiency anemia has been a concern in the Gaza Strip before the war [25, 26]. We calculated the energy contributed by protein and fat in food as 4 and 9 kcal per grams of protein and fat respectively.

We further categorized food items according to food groups (Grains, Legumes, Vegetables, Fruit, Oils, Meat poultry and fish, Dairy products and eggs, Sweets, Snacks, Sugar sweetened beverages, Other- see Supplementary Table 1). We also estimated the proportional weight of each food group within ready meals and food parcels according to their content (Supplementary Table 4). Thus, we were able to estimate the proportional weight of different food groups from different food categories per month.

Table 1 details our calculations of content, weight adjustment and nutritional value of each of the supply categories in our analysis.

**Table 1:** Supply categories included in the analysis and nutritional value

Supply category	Calculations of nutritional value	Weight adjustment factor		Protein (gr/100g adjusted weight)	Energy (kcal/100 g adjusted weight)	Fat (gr/100 g adjusted weight)	Iron (mg/100g adjusted weight)	
Specific food commodities	USDA’s food composition data	Adjustment for 15% loss.	Please see supplementary Table 1					
Standardized parcels:	The IFRC food parcel	Adjustment for 40% loss (Supplementary Table 2).		8.4	357	13.8	2.2	
	OXFAM Food Parcel			13.7	265.8	7.2	4.8	
	UNRWA Food Parcel			10.2	394.5	16.14	2.4	
	WFP Parcels			13.9	307.5	6.8	2.4	
	WCK Food Parcel			9.7	225.0	7.5	1.5	
	Other (average of other standardized parcels)			10.7	292.4	10.0	2.5	
Non-standardized parcels	The nutritional value was calculated as the weighted mean of all foods supplied as specific food commodities in all shipments throughout 7 months (Supplementary Table 1).	Weight was adjusted by 15% loss.		10.5	313	4.7	2.6	
Standardized parcels (mixed)	We used the nutritional content of the IFRC standardized food parcel (Supplementary Table 2).	Food weight was assumed as 50% of the supply weight, Further adjustment was made for 40% loss.		8.4	357	13.8	2.2	
Non-standardized parcels (mixed)	The nutritional value was calculated as the weighted mean of all specific food commodities in all shipments throughout 7 months (supplementary Table 1).	Food weight was assumed as 50% of supply weight, with additional adjustment for 15% loss.		10.5	313	4.7	2.6	
Ready meals	The nutritional value of a standard main meal (Supplementary Table 3).	Weight was adjusted for 15% loss.		8.0	204	8.4	1.5	
Foods for infants and toddlers								
Complementary food for infants > 6 months of age (mashed vegetables)	Food labels provided by the commercial international brand.	Weight was adjusted for 15% loss.		1.0	40.0	0	0.6	
Infant nutritional supplementation	Food labels provided, (IFRC catalogue) [27].			13.4	535.0	13	45	
Infant formula	Food label of a commercial international brand for stage 2 + 3 formulas.			15.5	426.0	11	10	

The Raw data obtained from COGAT registry, as of August 2024 and used in this analysis is available in supplementary Table 5.

### Per capita analysis of nutrients supplied to the Gaza strip

We calculated the total energy (in kcal), protein (in grams and as a percentage of total kcal), fat (in grams and as a percentage of total kcal), and iron (in milligrams) delivered across the borders into Gaza each month. Based on the population size of Gaza as reported by the Gaza Central Bureau of Statistics (*n* = 2,226,544 ) [28], we calculated the average amount of nutrients per person per day. This per capita analysis could then be compared with the needs of the Gazan population as a whole, based on the consensus standard of crisis affected populations’ dietary needs, the Sphere Standards “Essential concepts in food security and nutrition”, presented in Sphere Handbook Appendix 6: Minimum Population Requirements [29]. These scientific standards, drawn from 25 years of experience, provide practical guidelines and establish minimum humanitarian standards for addressing food security and nutrition.

### Sensitivity analysis

For sensitivity analysis and to avoid potential information bias, we used a stringent food-loss factor of 30% (compared to the acceptable 15% loss), to evaluate the ability of shipments to deliver the dietary needs of the Gazan population.

Furthermore, the age distribution of the Gazan population has a very high proportion of children. Accordingly, we performed an additional stringent, non-standard analysis to calculate the theoretical age-adjusted energy, protein, and iron supply [30], required by a healthy population with the Gazan age distribution, including a predominantly higher proportion of children. Daily per capita amounts of these nutrient requirements were calculated (supplementary table 5). Note that these are population-based guidelines and are not tailored for individuals or groups who are ill or for repletion of deficient individuals. Actual dietary needs might be higher.

### Regional availability of food

The Gaza strip is divided into the North of Gaza and Gaza governorates in the north, the central Deir el Balah and Khan Yunis governorates, and the Rafah governorate in the south. The Al-Mawasi humanitarian zone is located along the coast of the western Rafah and Khan Younis governorates. To address the concerns for the food availability of the Gazans remaining in the northern governorates we assessed the available data on the number of trucks and weight of food dispatched, according to a separate registry of trucks traveling internally from the southern crossing point to the north of Gaza coordinated with COGAT (supplementary material - Raw data). We also accounted for trucks entering from the north crossing points, and food air drops as destined to the north of Gaza. All remaining trucks entering from the southern crossing points were considered as destined to the south and center of Gaza. We could thus account for the number and weight of the trucks by destination – north vs. south and central Gaza. Content of the trucks crossing the strip from south to the northern governorates were not described so we could not assess their nutritional values.

### Stability of food supply

The COGAT database enabled us to calculate the amount and nutritional content of food delivered to the Strip from all sources and routes on a daily basis. These amounts were summed to weekly supply, and the weekly mean per capita supply was calculated, by dividing the total kcal provided to the Gaza Strip each week, between 1/1/24 and 27/7/24, by the total population number and by 7 (days of a week). The stability of weekly aid delivery was expressed as the distribution of the weekly mean supply of daily-per-capita energy. This includes the number of days during each month in which border crossings were closed and therefore food was not delivered (normally crossings are closed on Saturday).

## Results

The COGAT registry records a total of 28,734 trucks and airdrops supplied to Gaza between January and July 2024, conveying food weighing 478,229 tons. Between January and April, with the exception of February, the amounts of food delivered to the Gaza Strip increased and remained relatively stable until July. In February, when aid delivery from the Kerem Shalom crossing point was reduced, airdrops were initiated. In March, the Ashdod port in Israel was opened briefly for the transfer of aid to Gaza, and in April the northern Erez land crossing opened. After the Rafah crossing was closed by Egypt in early May, two additional land crossings were opened, and the US military established a Joint Logistics Over-the-Shore pier (JLOTS) to allow aid to be delivered directly to Gaza by sea (Table 2). Although JLOTS delivered approximately 9,000 tons of aid by late June, it was shut down in July due to repeated operating difficulties in high seas. Subsequent aid by sea to northern Gaza was supplied via the Israeli port of Ashdod and the Erez Crossing [31].

The mean number of deliveries crossing per month was 4,104 (137 per day), carrying 68,318 tons of food aid per month. The number of deliveries increased by a mean of 264 deliveries per month, increasing food weight by a mean of 5,275 tons every month from January to July 2024. About 42.2% by weight of the aid supplied to the Gaza Strip was delivered by UN humanitarian aid agencies, 14.8% by other humanitarian agencies, 7.6% by foreign states and 35.0% from the private sector. After adjusting for packaging and other non-food weights, the proportional weight of shipments indicates that standardized food parcels, and specific food commodities account for most of the food supply (Table 2). Noticeably, the share of food donated by UN agencies gradually decreased between April and July, from 51% of all food delivered in April, to 22% in July (Table 2, Supplementary Fig. 1). This was followed by a decrease in the share of food delivered in standardized food parcels (from 27.4% in April to 5.8% in July) and an increase in non-standardized food parcels (from 2% in April to 27% in July) (Table 2).

**Table 2:** Food supplied to the Gaza Strip between January-July 2024

	January		February		March		April		May		June		July	
Crossing points or routs of entry for food														
Operating crossing points ^a^	Kerem Shalom Nitzana\Rafah		Kerem Shalom Nitzana\Rafah **Airdrops**		Kerem Shalom Nitzana\Rafah Airdrops		Kerem Shalom Nitzana\Rafah Airdrops **Erez** ^b^		Kerem Shalom Nitzana\Rafah^c^ Airdrops Erez **JLOTS**		Kerem Shalom Airdrops Erez JLOTS		Kerem Shalom Airdrops Erez	
Operating aid delivery routes ^a^	Jordan Egypt		Jordan Egypt		Jordan Egypt **Ashdod port**		Jordan Egypt Ashdod port **Israel**		Jordan Egypt Ashdod port Israel **Judea&Samaria**		Jordan Egypt Ashdod port Israel Judea&Samaria		Jordan Egypt Ashdod port Israel Judea&Samaria	
Summary of food delivered														
Total deliveries (trucks and airdrops)	3,425		2,145		3,681		4,904		5,275		4,295		5,009	
Total weight (Tons)	52,293		30,290		61,330		83,587		94,884		71,903		83,942	
Food by donor (Tons, % of monthly total)														
UN aid agency	29,074	55.6%	14,550	55.6%	30,353	49.5%	43,207	51.7%	39,116	41.2%	27,297	38.0%	18,514	22.1%
International aid agency	12,173	23.3%	10,811	23.3%	14,755	24.1%	23,088	27.6%	7,024	7.4%	2,733	3.8%	917	1.1%
Nationalities	3,493	6.7%	1,194	6.7%	5,412	8.8%	12,806	15.3%	5,296	5.6%	6,548	9.1%	2,113	2.5%
Private sector	7,553	14.4%	3,734	14.4%	10,810	17.6%	4,487	5.4%	43,448	45.8%	35,325	49.1%	62,398	74.3%
Food by crossing point and destination (Tons, % of monthly total)														
Nitsana	19,790	37.8%	10,368	34.2%	17,605	28.7%	21,661	25.9%	2,857	3.0%	0	0.0%	0	0.0%
Kerem shalom – destined to south and center	0	0.0%	0	0.0%	8,380	13.7%	34,910	41.8%	7,640	8.1%	7,641	10.6%	0	0.0%
Kerem shalom – destined to north	32,503	62.2%	19,835	65.5%	33,850	55.2%	25,113	30.0%	63,197	66.6%	52,607	73.2%	69,030	82.2%
Erez	0	0.0%	0	0.0%	0	0.0%	0	0.0%	19,954	21.0%	9,941	13.8%	14,877	17.7%
Air drops	0	0.0%	86	0.3%	1,496	2.4%	1,904	2.3%	611	0.6%	103	0.1%	35	0.0%
JLOTS	0	0.0%	0	0.0%	0	0.0%	0	0.0%	626	0.7%	1,611	2.2%	0	0.0%
Food by supply type (Tons, % of monthly total)														
Specific food commodities	27,238	52.1%	14,707	48.6%	39,264	64.0%	56,105	67.1%	78,064	82.3%	49,772	69.2%	55,696	66.4%
Standardized food parcels	23,759	45.4%	15,130	50.0%	19,104	31.1%	22,906	27.4%	10,904	11.5%	7,885	11.0%	4,907	5.8%
Non-standardized food parcels	523	1.0%	83	0.3%	349	0.6%	1,679	2.0%	5,202	5.5%	13,689	19.0%	22,653	27.0%
Cooked meals	327	0.6%	218	0.7%	2,422	3.9%	2,639	3.2%	510	0.5%	476	0.7%	166	0.2%
Infant food	259	0.5%	29	0.1%	119	0.2%	60	0.1%	68	0.1%	9	0.0%	455	0.5%
Standardized food parcels (Mixed)	180	0.3%	123	0.4%	54	0.1%	122	0.1%	0	0.0%	0	0.0%	0	0.0%
Non-standardized food parcels (mixed)	6	0.0%	0	0.0%	19	0.0%	77	0.1%	136	0.1%	72	0.1%	66	0.1%

^a^ Newly opened food sourcing and delivery routes are indicated in **Bold** font during the first month of operation

^b^ Erez crossing opened on 17\04\24

c Rafah crossing closed by Egypt on 5\5\24

d Adjusted weight accounting for food loss, as depicted in Table 1

**Fig. 1:**
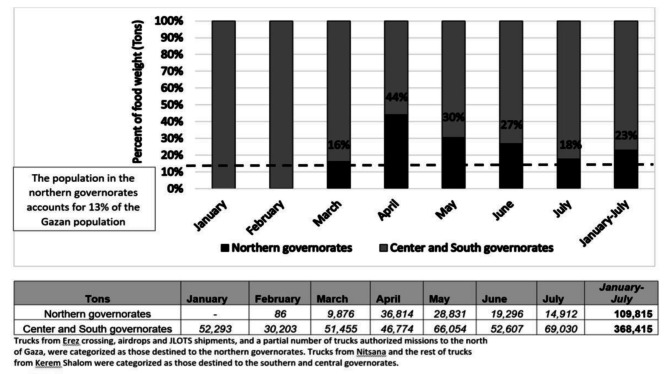
Monthly food delivered to the northern governorates, as a proportion of the total food weight entering the Gaza strip

To address aid supply to the northern parts of the Gaza Strip, we analyzed the weight of food deliveries entering the Strip from different crossing points, and from Kerem Shalom with different destinations. Some 300,000 people were assumed to be in the northern governorates of the Gaza Strip (13% of the population), compared to 1,926,544 people (87%) in the center and southern governorates of Gaza, according to recent IPC reports. This difference is reflected in the total number of trucks delivered during the 7 months analyzed: 5,109 trucks (17.7% of all truck delivered) containing 109,815 tons of food (22.9% of the weight of all food delivered), to northern Gaza, as compared to 23,625 trucks (82.2% of all truck delivered), carrying 368,415 tons (77.0% of the weight of all food delivered) delivered to the southern and central Gaza (Fig. 1).

**Fig. 2:**
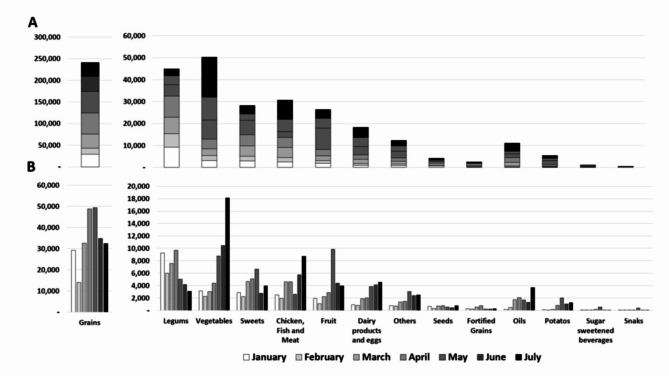
Amounts of food by food-group, delivered to the Gaza Strip in total **(A)** and by month **(B)**

The weight of food from different food groups (from all supply categories combined) demonstrates that shipments delivered a wide variety of foods to the Gaza Strip (Fig. 2). The most abundant food group delivered was Grains, with 241,434 tons over 7 months, and a mean increase of 533 tons per month. Next, Vegetables and legumes were delivered in high amounts (50,263 tons and 44,908 tons). However, while the mean monthly increase in vegetables was highest (2,497 tons per month), the amounts of legumes delivered decreased by a mean of -1,032 tons per month. Similar amounts of meat, chicken and fish, and sweets were delivered with 30,687 tons and 28,285 tons, respectively.

The nutrient content of food supplied to the Gaza Strip also increased over the period analysed, with the highest amount supplied in April. The mean monthly energy supply over 7 months was 3,004 kcal per capita per day. The mean amount of protein was 98.0 g per capita per day, comprising 13.2% of the daily kcal, the mean amount of fat was 61.2 g per capita per day (18.5% of daily kcal), and iron was 23.4 mg per capita per day. With the exception of February, food supply appeared compliant with Sphere standards each month during the months analyzed. We further performed a sensitivity analysis using a more stringent approach with a food-loss factor of 30%. With this conservative loss factor, energy and protein remained compliant with Sphere standards (Table 3, 4).

**Table 3:** Nutrient content of food supplied to the Gaza Strip, between January-July

Month	January	February	March	April	May	June	July	
Days per month	(31 days)	(28 days)	(31 days)	(30 days)	(31 days)	(30 days)	(31 days)	
**Adjusting by 15% food weight loss ** ^a^								
Average kcal / capita / day	2,424	1,508	2,919	4,167	3,886	2,893	3,232	
Average g protein / capita / day	91.5	55.2	100.2	142.4	119.2	91.4	85.9	
Average % of kcal from protein / capita / day	15.1	14.6	13.7	13.7	12.3	12.6	10.6	
Average g fat / capita / day	38.0	34.0	67.7	84.3	58.5	53.3	92.9	
% of kcal from fat / capita / day	14.1	20.3	20.9	18.2	13.6	16.6	25.9	
Average Mg iron / capita / day	21.2	12.3	23.1	34.0	29.0	22.6	21.6	
**Adjusting by 30% food weight loss ** ^a^								
Average kcal / capita / day	2,171	1,366	2,546	3,615	3,281	2,445	2,701	
Average g protein / capita / day	82.4	50.2	88.0	124.2	101.2	77.7	72.2	
Average % of kcal from protein / capita / day	15.2	14.7	13.8	13.7	12.3	12.7	10.7	
Average g fat / capita / day	36.5	31.9	60.2	75.2	50.8	45.8	77.7	
% of kcal from fat / capita / day	15.1	21.0	21.3	18.7	13.9	16.9	25.9	
Average Mg iron / capita / day	18.8	11.1	20.2	29.5	24.5	19.1	18.1	

^a^ Per capita analysis was performed by deviding the total amount of each nutrient by the population of Gaza (2,226,544 people) and the number of days per month

**Table 4:** Comparison between foods supplied to the Gaza Strip and Sphere standards for humanitarian aid supply to conflict-affected populations

^Average Individual Daily Requirements^	Sphere standards for humanitarian food supply ^a^	^Weight loss – 15%^		^Weight loss – 30%^	
		Average nutrients Supplied to Gaza^b^	Percent of Sphere standards met by supply	Average nutrients Supplied to Gaza^b^	Percent of Sphere standards met by supply
**Energy (Kcal/day)**	**2,100**	3,004	143%	2,589	123%
**Protein (g/day)**	**53**	98.0	185%	85.1	161%
**Protein (% as a proportion of total energy supply)**	**10%**	13.2%	132%	13.3%	133%
**Fat (g/day)**	**40**	61.2	153%	54.0	135%
**Fat (% as a proportion of total energy supply)**	**17%**	18.5%	109%	19.0%	112%
**Iron (mg/day)**	**32**	23.4	73%	20.2	63%

^a^ Sphere standards for humanitarian food supply - Population dietary needs according to Sphere standards were calculated by multiplying Sphere personal needs, with Gaza population size in 2023 (2,226,544 people)

^b^ Overall average was calculated as is the sum of each nutrient across 7 months, calculated according to the nutritional value of each food/supply category after adjustment of weight by 15% and − 30%, and divided by the sum of all days between January and July

### Sensitivity analysis

We calculated the age-adjusted energy, protein, and iron supply [32], required by a healthy population with the Gazan age distribution, which includes ~ 30% children under the age of 14, with appropriate dietary needs. The mean weighted theoretical daily requirements per capita for a population with such an age distribution were 1,934 kcal, 43 g protein, and 12 mg of iron (Supplementary Table 4). The food supplied to the Gaza Strip between January and July also exceeded these more stringent thresholds.

### Food availability stability analysis

**Fig. 3:**
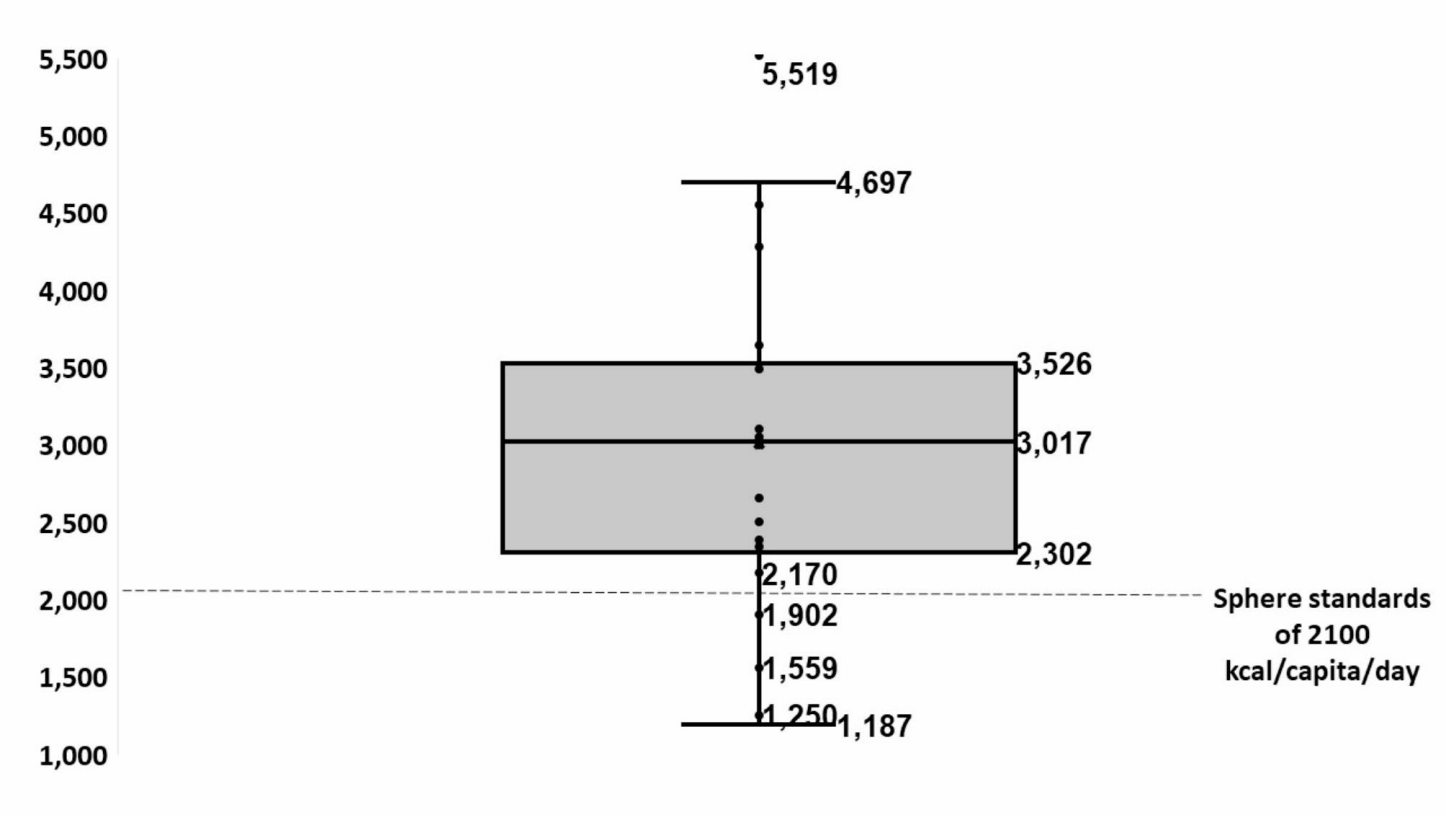
Supply stability, distribution of the weekly mean energy per capita, per day delivered to the Gaza Strip

The weekly mean daily energy availability per capita ranged between 1,187 kcal/capita/day (occurred between 18-25.2.24), and 5,519 (occurred between 16-23.5.24), with a mean of 3,002 ± 1054 kcal per day, and a median of 3,017 kcal per day (Fig. 3). Weekly mean daily energy availability fell below the Sphere standards of 2,100 kcal per day during 4 weeks in the 26-week study-period. These occurred during periods of intense fighting, during three weeks in February and the first week of May.

Other than that, during the study period there were fewer days in which the border crossings were closed to food delivery than before the war (e.g. on weekends and holidays): 4 closed days during January as was normal before the war, 6 days during February, 0 days during March, 2 days during April, 2 days during May, 0 days during June and 1 day during July.

## Discussion

This detailed analysis of the food per capita delivered to Gaza between January and July 2024, reveals that it meets the Sphere standards, even after applying stringent assumptions of food loss (e.g., food loss factors of 15 and 30%). In other words, the food transported across the borders into Gaza during this seven-month time period appears to have been sufficient to meet nutritional needs, on average, of the Gazan population. The lowest amount of food was supplied in February, when 1,508 kcal per capita per day were made available to the Gaza Strip. This period coincided with fierce combat operations and also efforts by some Israeli activists to halt the passage of humanitarian aid trucks via Kerem Shalom, claiming they were fueling Hamas [14, 33]. Food delivery stresses during this month may have served as an impetus for both the Food Security, Nutrition and Health Clusters alarms [6–8] and a COGAT response in the ensuing months, during which COGAT gradually opened new routes of entry into the strip and minimized the number of days in which borders were closed. This facilitated increased food deliveries into Gaza, with April and May showing the highest amounts of food supplied.

The amounts of Iron available in food supplied to the Gaza Strip appeared insufficient throughout the time period examined. This may possibly be an underestimate as we did not account for iron content in nutritional supplements with iron and other micronutrients that were included in shipments, or for iron-fortified foods delivered by aid organizations. It is noteworthy that the fourth most prevalent food group supplied throughout the time frame of the study was “sweets”. This food group contained cakes, cookies, candies, chocolate etc., which provide energy but contribute little to the supply of essential nutrients.

Though the nutritional value of the food supply changed by month, the mean amounts of daily per capita energy and protein content of food supplied to the territory remained above Sphere standards throughout all the months examined. The overall distribution of mean daily per capita energy of food supplied to Gaza by week showed overall consistent and stable results, with 4 exceptional weeks (3 of which were in February), in which mean energy content was estimated to have fallen below the Sphere standards. Another week in which energy supply was low was the first week of May, when Egypt closed the Rafah crossing point following Israel’s incursion into Rafah [34].

The findings of regional aid delivery reflect the month-by-month joint efforts made by aid agencies and COGAT to expand aid delivery routes and crossing points to the north of Gaza, motivated by concerns raised by international agencies about possible impending famine in this area. Initially, Kerem Shalom and Rafah land crossings, both in the south, were the only routes of entry of food to the strip. This constrained food distribution to the north from within the Gaza Strip. An IPC report in June 2024 noted the subsequent opening of the northern Erez and “96” land crossings, as well as air and sea delivery routes, as positive developments that improved aid available to northern Gaza. The IPC report also noted the steady increase in the delivery of food commodities to the north since March, such that the total quantity of food delivered in May could address the needs of the area’s 300,000 people [7]. This assessment concurs with our findings and those of the World Food Program Computer Assisted Telephone Interview (WFP CATI) household survey [7], indicating that in May, more than 80% of respondent households in northern Gaza received food aid.

The OCHA online dashboard [35] indicates that during January-September 2023, in the period preceding the war, the average daily rate of trucks entering Gaza was 321 (87,707 in total over 273 days), of which 100 per day were carrying food (27,434 trucks). According to UNRWA’s dashboard [36], which provides information regarding UN agency aid alone, the average number of trucks carrying food into Gaza daily increased from 55 in November 2023 to 97 in January, and 118 in March 2024 - which agrees with our analysis of COGAT data, and resembles passage of trucks entering Gaza before the war.

Thus, the evidence at hand is not consistent with claims that food aid delivery was deliberately restricted from entering Gaza during the period included in the study. However, any analysis of the food security crisis in Gaza should distinguish between food supplied to Gaza and downstream factors that may be hindering aid from reaching the civilian population. Food insecurity in Gaza has more likely been related to difficulties in reliably distributing, storing and assuring access to food aid once inside Gaza under conditions of war [18]. Numerous factors have reportedly hindered efforts of aid agencies to increase the amount of aid reaching crisis-affected people. These include security risks to aid workers [37, 38], the theft, looting, and hoarding of food and other supplies [39–41], and attacks against soldiers at humanitarian aid crossing points and corridors [42–44]. Damaged infrastructure, lack of aid workers, trucks, parts, and fuel have also impeded aid missions. During the war, damage to local agricultural production capacity and food system infrastructure, access restrictions to fishing areas, the upending of livelihoods, and mass displacement, have also contributed to the difficulties Gazans have faced reaching food [45]. Another significant challenge is that those Gazans who did not evacuate war zones have been harder to reach, and there has been a general uncertainty as to their numbers and actual places of shelter. Provision of aid to people residing in combat zones was understandably often restricted by aid agencies to reduce risk to aid workers. The extent and impact of these factors in Gaza should be addressed in future research.

Following projections of famine in earlier IPC reports [6, 8], and successes in subsequently transporting more food into the territory, a report from June stated that “In contrast with the assumptions made for the projection period (March – July 2024), the amount of food and non-food commodities allowed into the northern governorates increased. Additionally, the response in the nutrition, water sanitation and hygiene (WASH) and health sectors was scaled up” [7]. This is consistent with our findings.

The study has significant strengths including its foundation on systematically and comprehensively recorded data, compiled by COGAT since the end of December, from approved and completed food-aid consignments transferred across the border by UN agencies, State and humanitarian donors, and by private sector actors. This registry is more comprehensive than the UN databases of UNRWA, WFP and OCHA, which reflect only a part of the aid flow. Detailed analysis of the gaps and differences between UN and COGAT records reflect different methods of counting trucks, recording their contents and estimating shipment weights. For instance, the UNRWA dashboard largely lists the number of aid pallets rather than weights. UNRWA also only reports UN trucks entering Gaza via Rafah and Kerem Shalom land crossings that are managed by UNRWA. Trucks from other donors, from the private sector, or entering via other routes are incompletely covered by the UN database. Furthermore, UNRWA registers the trucks upon arrival at their warehouse, and thus may fail to account for trucks supplied to Gaza that are subsequently looted or stolen during transit [46]. A working paper published by Rosen and Nitzan described discrepancies of thousands of trucks listed in the COGAT database but absent from UNRWA records [47]. The June IPC report that compares COGAT, WFP, FEWSNET and OCHA data, clearly illustrates under-reporting of the food supply by the UN agencies (pp. 10–11, Fig. 5a and b) [7]. The report’s authors acknowledge that inconsistent methods of recording shipments by the different actors make it difficult to interpret the large discrepancies in the reported data. Therefore, they assessed trends in aid flow, instead of trying to reconcile absolute quantities, and concluded that the different databases describe consistent trends: “Between March and the end of April, the supply of food commodities in the northern governorate… the southern and middle governorates steadily increased according to many sources, despite differences in the absolute figures”. The consistency of the trends lends confidence in our findings.

Our study is the first detailed attempt to estimate the nutritional adequacy of the food supplied to the Gaza Strip during the war. It was reassuring that the mean food supply from January through July 2024 met the Sphere standards which take into account the dietary requirements of all age groups and both sexes, including pregnant and breastfeeding women, and the potential conflict-related dietary needs of the population [48]. Nevertheless, the gradual decline in the share of food delivered by UN agencies from 51% of all food delivered in April, to 22% in July, the causes of which have not yet been studied, and the increase in the reliance on private sector food supplies, is a cause for concern. This is due to the fact that commercial supplies do not include standardized food parcels, which are nutritionally balanced and include high amounts of protein-rich foods and iron fortified wheat flour. Additionally, food supplies from the private sector may be less accessible to the Gazan population, and especially to those with lower means to purchase it. This concern of food unaffordability is strengthened by reports of food prices rising, and market functionality being fragile, across the Gaza Strip [49]. On the other hand, an August 2024 report by the WFP Food Security Analysis of the Gazan Market Monitor reported that “.the entry of these commercial trucks has contributed to the recent stabilization of food prices in Deir Al Balah and Khan Younis.” [50]. 

Our study has several limitations: First, we only examined one dimension of food security, namely, national food availability at the borders, and the period covered is limited to January and July 2024. At the onset of the war, COGAT did not have a process in place to facilitate the delivery and documentation of a massive humanitarian intervention. As a result, reliable and systematic data did not become available until January. Weight of food on trucks was estimated based on a subsample of trucks with available data on exact weight. In trucks of mixed content, nutritional value was based on all foods delivered to the Gaza Strip, and not on actual truck content. The estimation and recording of gross consignment weight, the challenge of accounting for the dynamic regional distribution of displaced populations, and the necessity of estimating some of the shipment's weights, limits the precision of our estimates. Although our results are approximations, we have used stringent assumptions and extensive sensitivity analysis. Wwe further believe, that any potential bias would likely be non-differential because the estimates were consistent across all documented shipments. The stringent adjustment factors that we used to account for the potential overestimation of the food supply lend confidence that the absolute values we report are likely conservative. We did not evaluate access, utilization, and stability of food obtained by conflict-affected people in Gaza. The per capita food availability data do not imply the nutritional intake of individuals in the population. Rather, they indicate availability of adequate nourishment, provided that food commodities are equitably, efficiently, adequately and safely distributed. Furthermore, energy, protein, fat, and iron are imperfect proxies for total nutrient content of the supply. Because food storage and cooking conditions could not be verified, we could not reliably evaluate the actual micronutrient supply. Nevertheless, we chose to analyze iron as a reasonable proxy of foods with high nutrient density (both natural and fortified), and because of its importance for preventing iron-deficiency anemia. Systematic, quantifiable, objective, and verifiable examination of access to commercial and humanitarian food assistance is needed to augment the distribution of aid to conflict-affected people. Increased cooperation of all partners and shared efforts to overcome barriers between the Food Cluster with COGAT could help in this regard.

Last, our analysis only looked at the nutritional aspect of the humanitarian aid delivered to the Gaza Strip. Other aspects such as water, shelter and medical care should also be analyzed.

## Conclusions

The findings of this report, based on comprehensive transport documents and conservative estimations, indicate that food allowed to enter into Gaza throughout January-July 2024 was sufficient to meet minimum per capita daily nutritional needs of the territory’s two million conflict-affected persons, for energy, protein and fat, (but not iron), based on international humanitarian guidelines. However, while the adequacy of food crossing borders is crucial to food security, it cannot in itself ensure the food security of a population in a conflict zone.

We hope that despite all the obstacles, health professionals on both sides of the conflict, along with international partners, will be able to undertake efforts to work together, to improve the nutritional security of the people of Gaza. Enhanced coordination between the UN Food Security Cluster and COGAT, and shared efforts to overcome barriers, and the systematic, objective, and verifiable assessment of nutritional security (especially availability and distribution), are essential so that future donations can be tailored and distributed to meet the dynamic needs and circumstances of Gaza’s population, not only during but also after the war.

## Electronic supplementary material

Below is the link to the electronic supplementary material.

Supplementary Material 1

Supplementary Material 2

## Data Availability

Data is available upon reasonable request.

## Abbreviations

COGAT: Coordinator of Government Activities in the Territories
IDF: Israeli Defense Forces
UN: United Nations
NGO: Nongovernmental organizations
IPC: Integrated Food Security Phase Classification
UNRWA: United Nations Relief and Works Agency for Palestine Refugees in the Near East
ICRC: International Committee of the Red Cross
IFRC: International Federation of the Red Cross and Red Crescent Societies
USDA: United States Department of Agriculture
RDA: Recommended Dietary Allowance
