# Supplementary material for: Food supplied to Gaza during seven months of the Hamas-Israel war
Source: Isr J Health Policy Res. 2025 Feb 12;14:8. doi: 10.1186/s13584-025-00668-6 (PMC11818336; doi:10.1186/s13584-025-00668-6)

**Supplementary material**

**Supplementary table 1. Defined food commodities delivered to the Gaza Strip, food group categorization, nutritional value, and proportional weight in food commodities shipments**

| Food name | Energy (kcal/100 gr) | Protein (gr/100 gr) | Fat (gr/100 gr) | Iron (mg/100 gr) | Food group | Proportional weight (%) from all shipments |
| --- | --- | --- | --- | --- | --- | --- |
| Flour, wheat, all-purpose, enriched | 359 | 13.1 | 1.48 | 3.44 | Grains | 62.68 |
| Rice, white, long grain, unenriched | 359 | 7 | 1.03 | 0.14 | Grains | 1.82 |
| Pasta, dry, unenriched | 371 | 13 | 1.51 | 1.3 | Grains | 2.41 |
| Bread, pita, white, unenriched | 275 | 9 | 1.2 | 1.4 | Grains | 0.11 |
| Crackers, wheat, regular | 455 | 7.3 | 16.4 | 2.64 | Grains | 0.11 |
| Buckwheat groats, roasted, dry | 692 | 23.4 | 2.71 | 2.47 | Grains | 0.06 |
| Corn, sweet, yellow, canned | 61 | 2 | 0.77 | 0.36 | Grains | 0.07 |
| Corn meal | 371 | 8.8 | 1.2 | 1 | Grains | 0.02 |
| Oat Bran | 728 | 34 | 7.97 | 8.07 | Grains | 0.01 |
| Semolina | 720 | 26 | 1.05 | 1.23 | Grains | 0.1 |
| Bulgur, dry | 342 | 12.3 | 1.33 | 2.46 | Grains | 0.05 |
| Barley | 139 | 2.2 | 2.74 | 1.28 | Grains | 0.54 |
| Energy bar, emergency ^a^ | 440 | 16 | 15 | 9 | Fortified Grains | 0.07 |
| Legumes, (Non specified) ^b^ | 354 | 23 | 2.8 | 6.76 | Legumes | 0.01 |
| Beans, white, mature seeds, raw | 333 | 23.4 | 0.85 | 10.4 | Legumes | 0.68 |
| Lentils, raw | 352 | 24.6 | 1.06 | 6.51 | Legumes | 0.44 |
| Chickpeas mature seeds, raw | 378 | 20.5 | 6.04 | 4.31 | Legumes | 0.54 |
| Peas, green, split, mature seeds, raw | 364 | 23.1 | 3.89 | 4.73 | Legumes | 0.08 |
| Sesame butter, tahini | 592 | 17.4 | 53 | 4.42 | Seeds | 0.13 |
| Peanuts, all types, raw | 567 | 25.8 | 49.2 | 4.58 | Seeds | 0.1 |
| Seeds, sesame seeds, whole, dried | 573 | 17.7 | 49.7 | 14.6 | Seeds | 0.02 |
| Oil, canola | 884 | 0 | 100 | 0 | Oils | 2.26 |
| Chicken, broilers or fryers, meat and skin, raw | 215 | 18.6 | 15.1 | 0.9 | Chicken, Fish and Meat | 1.33 |
| Beef, cured, corned beef, canned | 250 | 27 | 14.9 | 2.08 | Chicken, Fish and Meat | 0.27 |
| Fish, cod, Pacific, raw | 69 | 15.3 | 0.41 | 0.16 | Chicken, Fish and Meat | 0.78 |
| Fish, tuna, light, canned in oil | 198 | 29.1 | 8.21 | 1.39 | Chicken, Fish and Meat | 0.22 |
| Beef, variety meats and by-products | 276 | 15 | 23.5 | 5.67 | Chicken, Fish and Meat | 1.18 |
| Cookies, butter, commercially prepared, unenriched | 467 | 6.1 | 18.8 | 0.29 | Sweets | 0.39 |
| Cake, sponge, commercially prepared | 290 | 5.5 | 2.7 | 2.72 | Sweets | 0.38 |
| Candies, halavah, plain | 469 | 12.5 | 21.5 | 4.53 | Sweets | 0.09 |
| Sweets, (Non specified) ^b^ | 355.16 | 6.45 | 15.26 | 2.10 | Sweets | 0.01 |
| Candies, milk chocolate | 535 | 7.65 | 29.7 | 2.35 | Sweets | 0.02 |
| Biscuit | 370 | 7 | 18.9 | 2.76 | Sweets | 0.54 |
| Rahat Lukum dessert | 448 | 9 | 24 | 0 | Sweets | 0.01 |
| Jams and preserves | 278 | 0.37 | 0.07 | 0.49 | Sweets | 0.02 |
| Sugars, granulated | 387 | 0 | 0 | 0.05 | Sweets | 3.02 |
| Honey | 304 | 0.3 | 0 | 0.42 | Sweets | 0.05 |
| Snacks, potato chips, plain, salted | 532 | 6.39 | 34 | 1.28 | Snacks | 0.12 |
| Snack, peanut butter | 534 | 17 | 30 | 8 | Snacks | 0.01 |
| Cheese, pasteurized processed cheese | 310 | 15.6 | 23.9 | 1.3 | Dairy products and eggs | 0.65 |
| Milk, fluid, 3% fat, without added vitamin A and vitamin D | 42 | 3.37 | 3 | 0.03 | Dairy products and eggs | 1.31 |
| Dairy products (Non specified) ^b^ | 152.75 | 6.22 | 11.86 | 0.36 | Dairy products and eggs | 0.25 |
| Sour cream, regular | 198 | 2.44 | 19.35 | 0.07 | Dairy products and eggs | 0.02 |
| Butter, without salt | 717 | 0.85 | 81.1 | 0.02 | Dairy products and eggs | 0.02 |
| Commercial powdered milk | 500 | 26 | 26 | 0.0 | Dairy products and eggs | 0.07 |
| Yogurt, plain, whole milk | 61 | 3.47 | 3.25 | 0.05 | Dairy products and eggs | 0.08 |
| Chocolate-flavor beverage mix for milk, powder | 89 | 3.27 | 3.17 | 0.03 | Dairy products and eggs | 0.05 |
| Dates, medjool | 277 | 1.81 | 0.15 | 0.9 | Fruit | 0.68 |
| Oranges, raw, with peel | 63 | 1.3 | 0.3 | 0.8 | Fruit | 0.15 |
| Clementine, raw | 47 | 0.85 | 0.15 | 0.14 | Fruit | 0.07 |
| Fruit (Non specified) ^b^ | 104.45 | 1.31 | 0.37 | 0.50 | Fruit | 3.84 |
| Lemon peel, raw | 47 | 1.5 | 0.3 | 0.8 | Fruit | 0.15 |
| Melons, cantaloupe, raw | 38 | 0.82 | 0.18 | 0.38 | Fruit | 0.05 |
| Apple, raw | 52 | 0.26 | 0.17 | 0.12 | Fruit | 0.11 |
| Bananas, ripe and slightly ripe, raw | 98 | 0.74 | 0.29 | 0.4 | Fruit | 0.02 |
| Pomegranates, raw | 83 | 1.67 | 1.17 | 0.3 | Fruit | 0.02 |
| Watermelon, raw | 30 | 0.61 | 0.15 | 0.24 | Fruit | 0.21 |
| Bananas, raw | 89 | 1.09 | 0.33 | 0.26 | Fruit | 0.13 |
| Guavas, raw | 68 | 2.55 | 0.95 | 0.26 | Fruit | 0.01 |
| Avocados, raw | 160 | 2 | 14.7 | 0.55 | Fruit | 0.02 |
| Tomato, raw | 22 | 0.7 | 0.42 | 0.1 | Vegetables | 0.01 |
| Onions, raw | 40 | 1.1 | 0.1 | 0.21 | Vegetables | 0.74 |
| Carrots, raw | 41 | 0.93 | 0.24 | 0.3 | Vegetables | 0.04 |
| Vegetables (Non specified) ^b^ | 40.5 | 1.01 | 0.17 | 0.25 | Vegetables | 5.15 |
| Garlic, raw | 149 | 6.36 | 0.5 | 1.7 | Vegetables | 0.05 |
| Potatoes, raw | 58 | 2.57 | 0.1 | 3.24 | Potatoes | 1.49 |
| Pickles, cucumber | 14 | 0.48 | 0.43 | 0.23 | Vegetables | 0.03 |
| Egg, whole, raw, fresh | 143 | 12.6 | 9.51 | 1.75 | Dairy products and eggs | 1.23 |
| Syrup, fruit flavored | 261 | 0 | 0.02 | 0.03 | Sugar sweetened beverages | 0.01 |
| Orange juice | 47 | 0.73 | 0.32 | 0.06 | Sugar sweetened beverages | 0.15 |
| Cola Beverages | 42 | 0 | 0.25 | 0.02 | Sugar sweetened beverages | 0.03 |
| Sugar sweetened beverages (Non specified) ^b^ | 44.5 | 0.36 | 0.28 | 0.04 | Sugar sweetened beverages | 0.16 |
| Tomato products, canned, sauce | 24 | 1.2 | 0.3 | 0.96 | Vegetables | 0.21 |
| Instant soup, noodle | 25 | 1.03 | 0.44 | 0.16 | Others | 0.19 |
| Soup, beef broth or bouillon, powder, dry | 213 | 16 | 8.89 | 1 | Others | 0.01 |
| Coffee |  |  |  |  | Others | 0.56 |
| Tea |  |  |  |  | Others | 0.07 |
| Multivitamin |  |  |  |  | Others | 0.13 |
| Salt |  |  |  |  | Others | 0.6 |
| Seasoning agents |  |  |  |  | Others | 0.44 |

Nutritional values of foods were obtained from the USDA database: <https://fdc.nal.usda.gov>

^a^ Nutritional values were obtained from the ICRC catalogue of food: <https://itemscatalogue.redcross.int/relief--4/food--5/nutrition-specialised-products--86/super-cereal-plus--FNUTSUPC01.aspx>, <https://itemscatalogue.redcross.int/relief--4/food--5/nutrition-specialised-products--86/emergency-food-ration-bar--FNUTEFRA01.aspx>, <https://itemscatalogue.redcross.int/relief--4/food--5/canned-food--15/ready-meal-canned--FCANMENU.aspx>

^b^ Nutritional values calculated as mean values of all other foods in the food group

**Supplementary table 2. Nutritional composition of Standardized food parcels**

| **Iron (mg)** | **Fat (gr)** | **Protein (gr)** | **Energy (kcal)** | **Weight (gr)** | **Food content per parcel** |
| --- | --- | --- | --- | --- | --- |
| IFRC food parcel | | | | | |
| 0 | 0 | 0 | 3,870 | 1,000 | SUGAR, white, 1kg |
| 0 | 0 | 0 | 0 | 1,000 | SALT, iodized edible, 1kg |
| 0 | 0 | 0 | 0 | 88 | YEAST, dried, package 11 gr |
| 4.1 | 24.6 | 87 | 594 | 300 | FISH, canned, sardines, veg oil, 150g |
| 13 | 15.1 | 130 | 3,710 | 1,000 | PASTA, durum wheat meal, 1kg |
| 13 | 15.1 | 70 | 3,590 | 1,000 | RICE, white, long grain, irri6/2, 1kg |
| 0 | 790 | 0 | 6,983.6 | 790 | OIL, rapeseed, 1liter |
| 104 | 8.5 | 234 | 3,330 | 1,000 | BEANS, white, small, 1kg |
| **2.18** | **13.8** | **8.4** | **357.3** |  | **Values per 100 g parcel ^a^** |
| **WFP Parcels** | | | | | |
| 172.4 | 241.6 | 820 | 15120 | 4000 | Canned Chickpeas/Chickpea Paste (400 g x10) 4.00 |
| 28 | 6.16 | 153.16 | 8316 | 2800 | Canned Fava Beans |
| 73.216 | 524.48 | 950.4 | 8800 | 3520 | Canned Chicken/ Beef (Only halal) |
| 44.88 | 13.2 | 140.36 | 10604 | 4400 | Canned Vegetables (Peas) |
| 67.95 | 322.5 | 187.5 | 7035 | 1500 | Halawa (30gx50) 1.50 |
| **2.38** | **6.83** | **13.88** | 307.49 |  | **Values per 100 g parcel ^a^** |
| UNRWA Food Parcel | | | | | |
| 79.04 | 6.46 | 177.84 | 2530.8 | 760 | Beans Can 380g |
| 0 | 105 | 97.5 | 1425 | 750 | Banda Vita cheese 250g |
| 2.688 | 0.84 | 3.36 | 67.2 | 280 | Saj Zlom Sauce 140 g |
| 0 | 1400 | 0 | 12376 | 1400 | Vegetable Oil 700 ml |
| 18.2 | 21.14 | 182 | 5194 | 1400 | Pasta 350 g |
| 26 | 30.2 | 140 | 7180 | 2000 | rice 1 kg |
| 0 | 0 | 0 | 0 | 750 | salt 750 g |
| 0 | 78 | 78 | 1500 | 300 | Milk Powder 300g |
| 14.1 | 178.2 | 45.9 | 3210 | 600 | Chocolate 300 g |
| 104 | 8.5 | 234 | 3330 | 1000 | White Beans 1 kg |
| 0.5 | 0 | 0 | 3870 | 1000 | sugar 1kg |
| 5 | 6 | 44 | 1855 | 500 | Grits 500g |
| 43.1 | 60.4 | 205 | 3780 | 1000 | Hummus I kg |
| **2.49** | **16.14** | **10.29** | **394.5** |  | **Values per 100 g parcel ^a^** |
| **OXFAM Food Parcel** | | | | | |
| 332.8 | 27.2 | 748.8 | 10656 | 3200 | Ful with tomato and oil, ready to eat (400g) easy open |
| 137.92 | 193.28 | 656 | 12096 | 3200 | Hummus (chickpeas) 400g easy open |
| 332.8 | 27.2 | 748.8 | 10656 | 3200 | Cooked White Beans Can (400g) easy open |
| 3.136 | 98.048 | 6.592 | 928 | 640 | Green Olives Can (640g) Pitted. |
| 21.98 | 38.15 | 2.94 | 406 | 350 | Olives, black, can 350 g, Pitted. |
| 56.576 | 405.28 | 734.4 | 6800 | 2720 | Luncheon Beef Can (340g) |
| 30.94 | 371 | 121.8 | 4144 | 700 | Tahini (700 g) |
| 0 | 2.1 | 1.5 | 1520 | 500 | Honey 500 g |
| 5 | 0 | 0 | 1665 | 500 | date syrup - 500g |
| 900 | 1500 | 1600 | 44000 | 10000 | high energy biscuits Box of 50pcs |
| 0 | 0 | 0 | 0 | 200 | Tea bags (200g) |
| 0 | 0 | 0 | 0 | 1000 | Zaatar - Thyme (1000 g) |
| 31.71 | 150.5 | 87.5 | 3283 | 700 | Halawa (700 g) (two pcs of 350g) |
| 4.5 | 0.75 | 9.05 | 1385 | 500 | Dates Pitted - 500g package |
| 13.3 | 2.55 | 16.95 | 1205 | 500 | Apricots, dried (500 g) |
| 8.95 | 1.25 | 16.5 | 1495 | 500 | Raisins, dried (500 g) |
| 10.425 | 61.575 | 218.25 | 1485 | 750 | Sardines, tinned (125g) easy open |
| 14.178 | 83.742 | 296.82 | 2019.6 | 1020 | Tuna, canned (170 g) easy open |
| 107.844 | 88.692 | 526.68 | 8299.2 | 2280 | Peas 380g , cooked canned easy open |
| 0 | 0 | 0 | 0 | 700 | Salt, iodized (700g) |
| **4.77** | **7.24** | **13.7** | **265.7** |  | **Values per 100 g parcel ^a^** |
| **WCK 2 day (A) Food Parcel** | | | | | |
| 1.68 | 0 | 1.2 | 1216 | 400 | Honey |
| 8 | 1.76 | 43.76 | 568 | 800 | Canned fava beans |
| 28.446 | 39.864 | 135.3 | 2494.8 | 660 | Canned chickpea paste |
| 3.28 | 4 | 15.6 | 512 | 800 | Canned sweet corn |
| 4.726 | 27.914 | 98.94 | 673.2 | 340 | Canned tuna fish |
| 7.072 | 50.66 | 91.8 | 850 | 340 | Canned beef |
| 0 | 1.6 | 5.6 | 488 | 800 | Fruit puree for kids (squeezy) |
| 6.3 | 1.05 | 12.67 | 1939 | 700 | Dates |
| 0 | 119.6 | 92 | 1444.4 | 460 | White cream cheese |
| 21.978 | 26.4 | 59.4 | 1914 | 660 | Toast |
| 0 | 0 | 0 | 0 | 50 | Zaatar |
| 17.68 | 212 | 69.6 | 2368 | 400 | Tahini |
| **1.5** | **7.5** | **9.7** | **225.7** |  | **Values per 100 g parcel ^a^** |

The content of food parcels were obtained from different humanitarian aid organizations. The nutritional contribution of each food item in the parcels was calculated based on the USDA database, in adjustment to its weight in the parcel.

**^a^** Nutritional values per 100g were calculated for gross dry food weight.

**Supplementary table 3. Nutritional values assigned to cooked meals**

| **Iron (mg)** | **Fat (gr)** | **Protein (gr)** | **Energy (kcal)** | **Weight (gr)** | **Estimated cooked meal composition** |
| --- | --- | --- | --- | --- | --- |
| 0.6 | 4.2 | 5.1 | 231.4 | 133 | White rice, cooked (1 cup) |
| 3.1 | 17.5 | 23.1 | 304.1 | 222 | Stewed, seasoned, beef with vegetables and potatoes (1 cup) |
| 0.4 | 1.0 | 0.0 | 20.1 | 30 | Matbuha, vegetable-based spread (4tbls) |
| 2.7 | 16.1 | 5.1 | 178.5 | 30 | Tahini (2tbls) |
| 0.8 | 0.7 | 5.5 | 165.0 | 60 | Pitta bread (1unit) |
| 0.0 | 3.0 | 2.0 | 139.9 | 33 | Baklawa (3 pieces) |
| **1.5** | **8.4** | **8.0** | **204.5** |  | **Values per 100 g ^a^** |

The content of the cooked meals in deliveries were estimated based on prior knowledge of the Ramadan evening meal, since most meals were donated during the month of Ramadan. The nutritional contribution of each food item in the parcels was calculated based on the USDA database, in adjustment to its weight in the parcel.

**^a^** Nutritional values were calculated per 100g gross dry food weight.

Supplementary table 4. Proportional weight of each food group within ready meals and food parcels

|  | Ready meals | WFP Parcels | WCK Food Parcel | UNRWA Food Parcel | OXFAM Food Parcel | The IFRC food parcel | | Standardized food parcel (all other donors)^a^ | Non-standardized parcels | Standardized parcels (mixed shipments) | Non-standardized parcels (mixed shipments) |
| --- | --- | --- | --- | --- | --- | --- | --- | --- | --- | --- | --- |
| Grains | 26% |  | 23% | 33% |  | | 32% | 16% | 68% | 16% | 68% |
| Fortified Grains | 12% |  |  |  |  | |  | 2% | 0% | 2% | 0% |
| Ready meals |  |  |  |  |  | |  |  |  |  |  |
| Legumes |  | 64% | 23% | 24% | 28% | | 16% | 28% | 2% | 28% | 2% |
| Oils |  |  |  | 2% | 2% | | 13% | 1% | 2% | 1% | 2% |
| Seeds | 6% |  | 6% |  | 2% | |  | 3% | 0% | 3% | 0% |
| Chicken, Fish and Meat | 44% |  | 11% |  | 11% | | 5% | 13% | 4% | 13% | 4% |
| Sweets | 6% | 9% | 6% | 14% | 27% | | 16% | 12% | 5% | 12% | 5% |
| Snacks |  |  |  |  |  | |  |  | 0% |  | 0% |
| Dairy products and eggs |  |  | 7% | 9% |  | |  | 3% | 4% | 3% | 4% |
| Fruit |  |  | 23% |  | 5% | |  | 6% | 5% | 6% | 5% |
| Vegetables | 6% | 27% |  | 12% |  | |  | 9% | 6% | 9% | 6% |
| Potatoes |  |  |  |  |  | |  |  | 1% |  | 1% |
| Sugar sweetened beverages |  |  |  |  |  | |  |  |  |  |  |
| Others |  |  | 1% | 6% | 26% | | 18% | 7% | 2% | 7% | 2% |

^a^ Standardized food parcels from all other donors, besides those specified

**Supplementary table 5. Gaza population size, 2023 size (Gazan CBS) and calculated dietary allowance**

| Daily nutrient supply requirements | | | Daily RDA per person^a^ | | | Population size, 2023 | Age group |
| --- | --- | --- | --- | --- | --- | --- | --- |
| Iron | Protein | Kcal | Iron | Protein | Kcal |  |  |
| 1,661,786 | 3,086,174 | 237,398,000 | 7 | 13 | 1000 | 237,398 | 0-3 |
| 2,960,360 | 5,624,684 | 414,450,400 | 10 | 19 | 1400 | 296,036 | 4-8 |
| 2,342,400 | 9,955,200 | 497,760,000 | 8 | 34 | 1700 | 292,800 | 9-13 |
| 3,630,224 | 16,475,632 | 558,496,000 | 13 | 59 | 2000 | 279,248 | 14-18 |
| 8,379,784 | 32,874,536 | 1,418,117,250 | 13 | 51 | 2200 | 644,599 | 19-45 |
| 3,867,593 | 11,817,644 | 537,165,625 | 18 | 55 | 2500 | 214,866 | 19-45 pregnant/lactating women ^b^ |
| 2,120,448 | 13,517,856 | 503,606,400 | 8 | 51 | 1900 | 265,056 | 46+ |
|  | | | | | | 2,230,003 | **Total population** |
| 24,962,594 | 93,351,726 | 4,166,993,675 | Per total population | | | Theoretical daily nutrient supply required by a healthy population with the Gazan age distribution | |
| 748,877,828 | 2,800,551,780 | 125,009,810,250 | Per capita | | |  |  |
| 12 | 43 | 1,934 | Per capita, accounting for 30% food loss | | |  |  |

^a^ RDA's were obtained from "Dietary Reference Intakes: Applications in Dietary Planning, Institute of Medicine of the National Academies, Washington DC, 2003:p 22"(30). These estimates are not appropriate for individuals or groups who are ill or for repletion of deficient individuals. Actual dietary needs are likely to be higher.

^B^ The number of pregnant and lactating women were added to the population size, calculated as the number of children aged 0-1 years

**Supplementary figure 1**. **Distribution of food source by month**


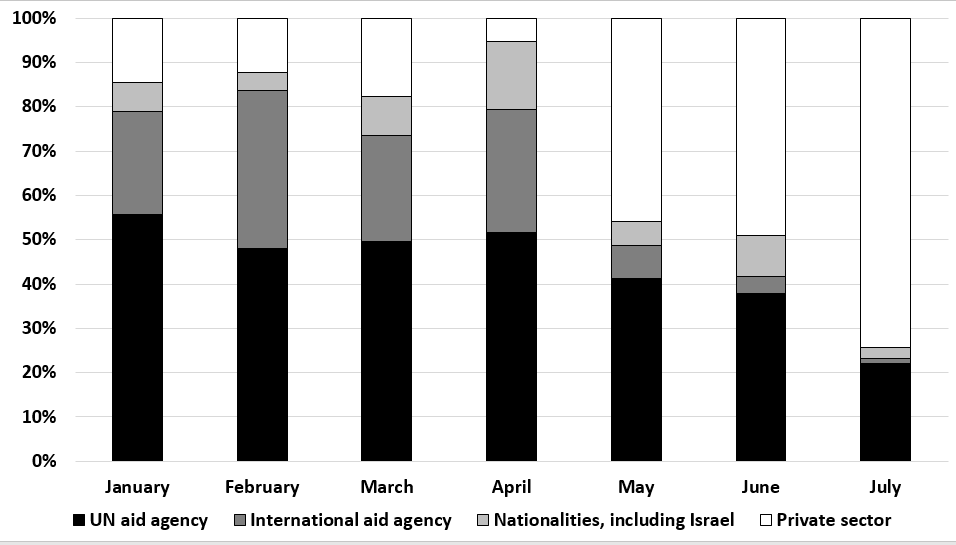

Supplement: Supplementary file 2 — Supplementary Material 2 [file 13584_2025_668_MOESM2_ESM.docx]
